# Experimental Analysis of Magnetic Focusing of the Plasma Arc of a Cutting Torch

**DOI:** 10.3390/ma18081811

**Published:** 2025-04-15

**Authors:** Martin Marek, Dejan Brkić, Pavel Praks, Tomáš Kozubek, Jaroslav Frantík

**Affiliations:** 1 ENET Centre, VSB - Technical University of Ostrava, 70800 Ostrava, Czech Republic; 2 Department of Technical Studies, College of Polytechnics Jihlava, 58601 Jihlava, Czech Republic; martin.marek@vsb.cz; 3 IT4Innovations, VSB - Technical University of Ostrava, 70800 Ostrava, Czech Republic; pavel.praks@vsb.cz (P.P.); tomas.kozubek@vsb.cz (T.K.); 4 Faculty of Electronic Engineering, University of Niš, 18000 Niš, Serbia

**Keywords:** plasma arc, magnetic focusing, cutting torch, microscopes, simulation software

## Abstract

This study aimed to verify the possibility of stabilizing and focusing a plasma column generated by a plasma cutter. The simulation performed by the COMSOL Multiphysics software is based on the actual configuration and geometry of the burner. This article presented a universal computational method based on FEM simulations, focusing on the deflection of the current of electrically charged particles in a magnetic field within the context of a plasma cutting torch. The simulations estimate the optimal shape and positioning of a focused electron beam for various magnetic lens positions and plasma stream energies, revealing that higher initial electron energies lead to a more even beam focus. Among the configurations tested, positioning the cathode 3 mm above the ring-shaped permanent magnet proved most effective, maintaining beam linearity and minimizing electron scattering, making it suitable for practical implementations.

## 1. Introduction

The objective of this study is to verify the possibility of stabilizing and focusing a plasma column generated by a plasma cutter using magnetic stabilization, with simulations exploring various magnet positions and configurations to optimize beam focus and minimize electron scattering [1,2,3,4]. The location of the permanent magnet at the mouth of the burner is a partial variable, especially in the axial height below the cathode. This focusing is also relevant to magnetic lenses in electron microscopes, although application in such devices requires significantly higher energy levels [5]. Focusing of the plasma cutting torch in this article is studied through modeling of the plasma arc by the COMSOL Multiphysics ver. 5.6 software [6,7,8,9].

Together with cutting by laser and oxygen, plasma arc cutting technologies are widely used in the industry due to their ability to effectively cut a large variety of materials [10,11,12,13,14]. Plasma modeling and numerical simulation serve as valuable tools for designing and optimizing arc plasma cutting devices [15,16].

The quality of the plasma arc cut depends on various process parameters, such as the cutting power, cutting speed, cutting height, plasma gas pressure, etc., which can be statistically analyzed. However, further research is necessary to establish a connection between the plasma arc simulation and reliable prediction of the cut quality [17,18,19,20,21,22,23].

Although plasma technologies are often used in waste gasification [24,25,26] and various recycling processes [24], these applications do not require precise focusing of a plasma torch. On the contrary, the expansion of the plasma torch is required for these applications. Additionally, magnetic focusing has many other applications, such as sensors for measuring the crankshaft angle of the main engine in ships [27]. Although this application is far beyond the scope of the study described in this article, it also uses the COMSOL Multiphysics ver. 5.6 software for modeling.

This article presented the first pilot study conducted in the Czech Republic that calculated the deflection of a plasma discharge (electric arc) using a concentrated magnetic field excited by a magnet. In this study, the plasma column area (discharge) was considered as a flow of charged particles (electrons). A realistic plasma torch model was used to simulate the trajectories and, consequently, the plasma deflection, employing FEM methods and the COMSOL Multiphysics environment. Furthermore, this analysis was a pioneer in considering several configurations of the permanent magnet positions and its pole attachments with respect to the plasma column (electro discharge). The deliberate use of a magnetic ring to focus/defocus the discharge in the plasma cutter nozzle is chosen because the electric discharge in the nozzle is relatively homogeneous and distributed over short distances in this configuration. This study clearly demonstrated the effects of the positions and configurations of the focusing magnet, including the impact on the initial energies of the electrons released from the electrode.

The aim of this study was to answer a straightforward scientific question: What levels of focus or defocus of the plasma beam of a plasma cutter can be achieved for specific configurations of the plasma column and magnetic field exciter in the form of a ring permanent magnet? After Section 1 with the introduction, this article is organized as follows: Section 2 provides a brief introduction to plasma and electric discharge physics. Section 3 presents an analysis of a specific type of plasma torch, including a computational analysis of plasma discharge deflection and focusing using a permanent magnet for various nozzle and magnet configurations. Section 4 summarizes the most important findings. Finally, Section 5 discusses conclusions and future research directions.

## 2. Fundamental Principles of Lens and Plasma Arcs Used for Cutting

Plasma cutting technology is partly based on the principle of electromagnetic lenses, in which the position of the plasma torch for material cutting is controlled by the electromagnetic field. Electromagnetic lenses are used for the deflection of electron beams and for the control of the direction of electrons. Thus, the same principles used for focusing through electron microscope lenses are used for the modeling presented in this article [28].

Generally, particles (electrons) are released from the surface of the negative electrode and pass through the collimator, a device which narrows a beam of particles or waves, which can be adjusted to remove stray electrons. A simple coil or ring magnet produces an axial magnetic field. This rotationally symmetric, inhomogeneous magnetic field results in a radial force acting on the off-axis electrons, causing these electrons to rotate about the axis. As they start to rotate (acquire spin) and move in a spiral trajectory, they undergo an increase in velocity, gaining a greater component perpendicular to the predominantly axial magnetic field. The parallel beam of electrons entering the lens then converges to a point. If the region affected by the magnetic field on the electrons is sufficiently limited, the coil or magnet functions akin to a “thin” convex lens.

The efficiency of plasma cutting depends not only on good focusing and navigation of the torch but also on other physical characteristics, especially the temperature of a plasma arc.

**Figure 1:**
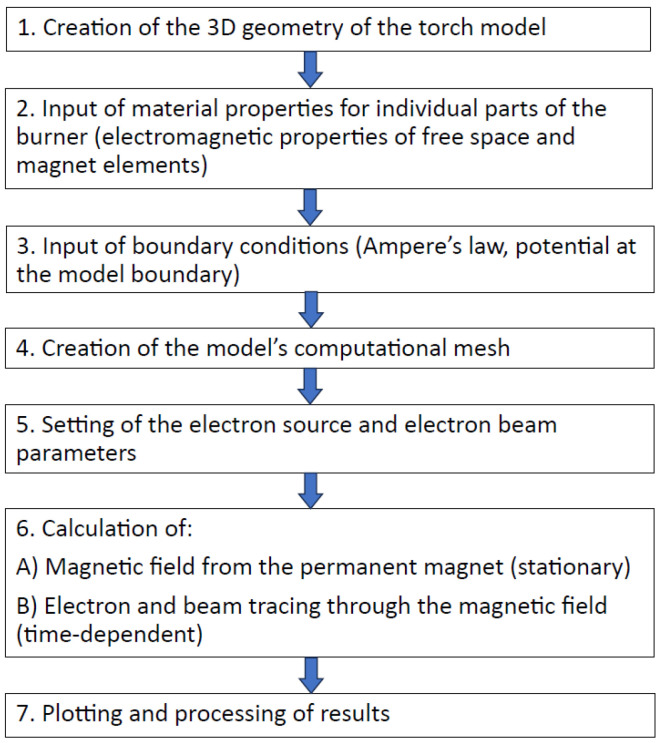
Roadmap of the design process for plasma cutting systems.

The general algorithm used in this article is given in Figure 1.

### 2.1. Electromagnetic Lens

Magnetic lenses are components that use magnetic fields to control and manipulate charged particles, such as electrons or ions, in various applications, including particle accelerators and electron microscopes. These lenses function similarly to optical lenses but use magnetic fields instead of glass to bend and focus particle beams [29], allowing for precise control and manipulation of particle trajectories.

The magnetic force on charged particles *F* [N], known as the Lorentz force, is given in Equation (1), while the focal length *f* [m] is generally given by the relation for the case of a cylindrical coil forming a lens as given in Equation (2).

(1) $$F=q\left(v\times B\right),$$

(2) $$f=K\cdot \frac{U}{i^{2}},$$

In Equations (1) and (2), *q* is the charge of a particle [C], *v* is the velocity of the particle [m/s], *B* is the magnitude of the magnetic induction at a given location [T], *K* is a coil size constant [dimensionless], *U* is accelerating voltage [V], and *i* is the magnitude of current through the coil [A].

**Figure 2:**
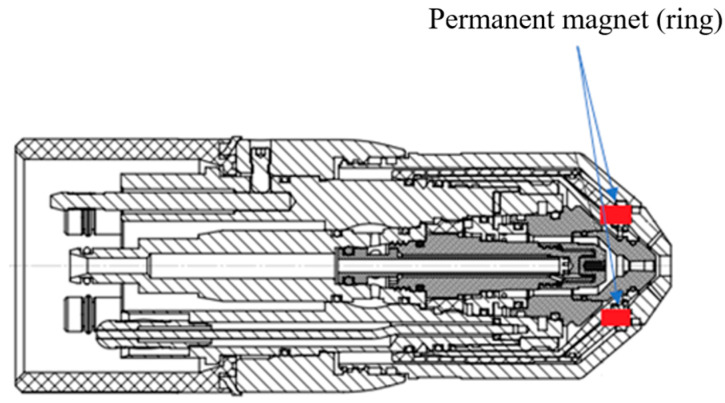
Schema of the used torch with the place of the permanent ring magnet.

The position of the permanent magnet of the torch used in this study is shown in Figure 2.

Schema of the used torch with the place of the permanent ring magnet (The basic parameters of the burner used for this study are: DC voltage—125 V, Direct current–260 A, Equivalent performance–32.5 kW, Plasma gas—O_2_ with Inlet pressure 5.5 bar and Inlet temperature 20 °C, Shielding gas—air with Inlet pressure 5.17 bar and Inlet temperature 20 °C, Coolant—70% + Distilled water 30% Propylene glycol with Inlet pressure 6.2 bar and Inlet temperature 20 °C, flow rate—minimally 2.3 L/min, nominal flow rate—3.5 L/min, cutting material—steel, thickness—25 mm, cutting speed—1685 mm/min, cutting height above the material—3.6 mm, plasma with the temperature at a distance from the nozzle mouth—2 mm at approx. 20,000–22,000 °C, 10 mm at approx. 18,000 °C, 15 mm at approx. 14,000 °C, 20 mm at approx. 10,000 °C).

**Figure 3:**
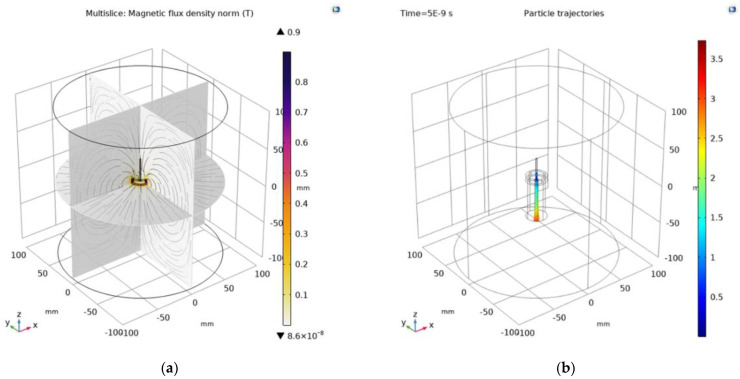
Magnetic flux and trajectories: (**a**) Magnetic field in the lens—magnetic induction; (**b**) beam particle trajectory—position.

The magnetic strength of the lens depends on the size of the coil, the number of turns and current, or the size and quality of the permanent magnet. Figure 3a shows magnetic flux, and Figure 3b shows typical trajectories of electrons as they pass through a coil. In general, the electrons are focused on a point along the z-axis.

In general, the focal length increases with higher electron energy (that is, with higher accelerating voltage) because the electrons’ high speed means they spend less time in the applied magnetic field. However, as the current of the lens coil or the size of the magnet increases, the magnitude of the excited magnetic field increases and, therefore, the electrons spiral in tighter orbits and bring the focal length closer.

### 2.2. Physics of the Cutting Arc

An electric discharge is a short–term physical phenomenon in which the insulator becomes a conductor of electric current due to the thermal processes taking place in it. In metal conductors, electrically charged particles are free electrons that are released and set in motion by the source. Conductors form electrodes in the discharges, near where the electron conductivity changes into ionic conductivity in the column of the discharge. Although the conductivity in the discharge column is called ionic, current conduction is mainly mediated by electrons. Depending on the size of the current and partly also on the electrodes, several types of electric discharges can be distinguished [30,31]; dark discharge, glow discharge, and arc discharge. The arc discharge is of interest for the study described in this article.

Macroscopically, the arc appears as a luminous, sharply defined gas formation between the electrodes. In the first approximation, it is considered symmetrical, but the axis of symmetry is not a straight line. The glowing body is formed by arc plasma. Plasma is heated to a relatively high temperature and is a good conductor of electricity.

In the areas of the electrodes, the potential rises very quickly in the areas of cathodic and anodic depletion. Cathodic and anodic loss have different sizes, and their value depends on the material of the electrodes.

An electric arc is a discharge burning in gas, capable of independent existence at any length of time (if it is not interrupted by a suitable intervention in its mechanism).

The main features of the arc are:

- The high temperature of the cathode spot (or the cathode in general) sufficient for the thermal emission of electrons;
- The current density of the cathode spot is of the order of tens of MA/m^2^;
- Small electrode losses;
- The small voltage between the electrodes (relative to the voltage of the source);
- Large current passing through the arc (greater than 1 A);
- Intense light emission from the discharge plasma and the electrodes.

According to the type of voltage in the circuit in which the arc burns, they are divided into arcs fed by direct current (DC) and arcs fed by alternating current (AC). The DC arc remains static, with the relationship between the voltage across the electrodes and the arc current referred to as the static characteristic, while the AC arc, which varies with time, is referred to as the dynamic characteristic. Even though AC is subject to time variation, it can maintain a stable quasi-stationary state while its parameters change following fluctuations in the electric current supply over time.

When an arc occurs, a transitional stage always occurs. During the formation of the arc, its plasma is thermally unbalanced. The glow discharge practically does not manifest itself in the transient stage because it can only exist at a pressure lower than the atmospheric pressure. The arc formation time is very short, in the order of 10^−6^ s. After formation, the plasma of the arc is in thermal equilibrium, which means that particles (electrons, ions, neutral atoms, molecules, etc.) have the same temperature.

#### 2.2.1. Arc Temperature

When an electric current passes through the plasma, heat is released, resulting in an increase in the temperature of the plasma. In a very short time after the formation of the arc, its plasma is heated to a high temperature. The temperature is not the same throughout the plasma cross-section. It is highest in the plasma axis and decreases towards the edges of the plasma referring to the temperature distribution depending on the radius as the radial temperature course. The temperature should be high enough to induce ionization processes.

The dependence of temperature *T* in the plasma axis on the current passing through an arc burning in the extinguisher with the compressed air is given in Equation (3).

**Figure 4:**
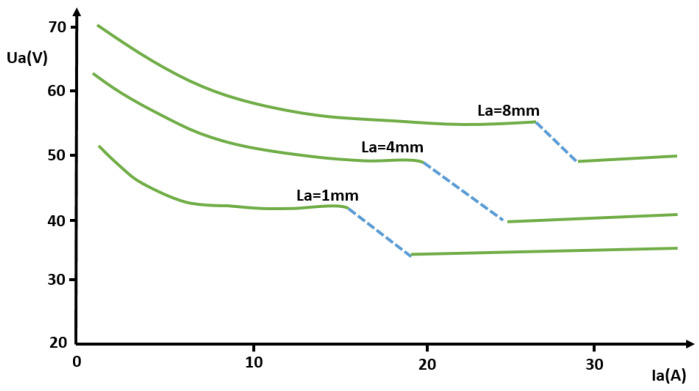
Characteristics of the arc (curves before the turbulent burning zone).

(3) T=30.885·Ia1.833·Ia+9400·ra223,

where *T* is temperature [K], *I_a_* is current through the arc [A] (which can be also extracted from the characteristic of the discharge given in Figure 4), and *r_a_* is the arc radius [m] [32]. This relation applies to current through the arc *I_a_* ≥ 800 A and radius of the arc 3 mm < r_a_ < 6 mm.

#### 2.2.2. Discharge Characteristic of the Arc

The Ayrton relation [33], given here in Equation (4), gives a correlation between the voltage across the electrodes, *U_a_* [V] and the current passing through the arc, *I_a_* [A]. It comprises a set of curves based on the different lengths of the arc, *L_a_* (given in Figure 4 [mm]). Material and dimensions of the electrodes, the type of the environmental gas, and its pressure also influence these characteristics through *α*, *β*, *γ*, *δ*, with Table 1 providing these constants of the Ayrton relation for copper and carbon in ambient air.

(4) $$U_{a}=\alpha +\beta \cdot L_{a}+\frac{\gamma +\delta \cdot L_{a}}{I_{a}},$$

where U_a_ is the voltage between the electrodes [V], *I_a_* is the current passing through the arc [A], La is the length of the arc, and *α*, *β*, *γ*, *δ* are constants depending on material, shape, and dimensions of the electrodes, as well as on the type and pressure of the gas in which the arc burns (*α* is the sum of electrode losses [V], while *β* is arc voltage drop [V/m]).

The Ayrton relationship is applicable to short arcs and relatively small currents.

## 3. Experiments and Simulation Results

This section gives a concise note related to the software COMSOL Multiphysics ver. 5.6 used for the performed simulation and then goes to the results. The results of physical field simulations consist of:

- Basic simulation with a permanent magnet alone and with a 3D torch together with a magnet;
- Main simulation with a reduced 3D burner model (with geometric and energy variations).

The required analysis was performed in the COMSOL Multiphysics simulation software [5], which is intended for solving various problems in physics. This package contains both basic modules for heat sharing, fluid flow, and electromagnetism and extension modules for both material libraries and modules for tracing charged particle trajectories, and finally a complex module for plasma simulation. However, this module is not included in the basic offer, and it is necessary to purchase it for a substantial additional fee. The program itself is user-friendly and scales on multi-core computing systems.

### 3.1. Basic Simulation

#### 3.1.1. Permanent Magnet Alone

In the first step, a basic verification simulation of the permanent magnet, itself placed in free space, was performed in the form of a 2D axisymmetric model.

**Figure 5:**
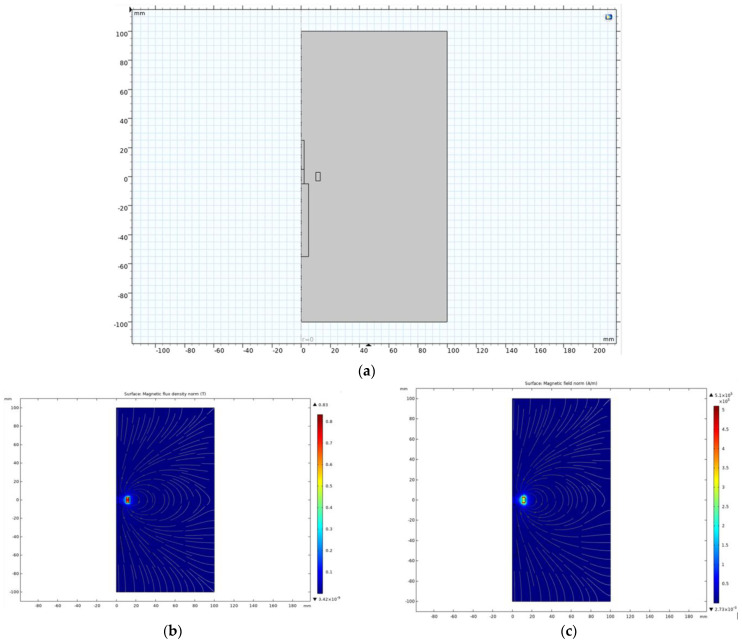
Permanent magnet placed in free space in the form of a 2D axisymmetric model: (**a**) 2D model of a permanent magnet in the air; (**b**) magnetic induction *B* (T); (**c**) magnetic field intensity *H* (A/m).

The permanent magnet’s dimensions were as follows: inner diameter of 20 mm, outer diameter of 26 mm, and magnet height of 6 mm. The material of the permanent magnet was NdFeB [34,35], parameters: *B_r_* = 1.05 T, *H_c_* = 838,000 A/m. The surrounding size is 10 cm on all sides. The implementation of the model is shown in Figure 5a, including the calculated distribution of the magnetic field in Figure 5b,c.

This simulation was checked with the calculation in the electromagnetic Ansys–Maxwell [36,37,38] simulator, and the agreement was very good. The simulation results show the magnetic field of a transversely magnetized permanent ring magnet. Part of the magnetic field is enclosed by the external surroundings of the magnet, and part of the magnetic flux is then enclosed by the inner cavity of the magnet. This magnetic field acting in the axis of the magnet then forms its own active field, which can act on the plasma arc and partially deflect or focus the charged particles forming the body of the arc.

#### 3.1.2. 3D Torch with Magnet

In this step, a 3D model corresponding to the geometry of the cutting torch, including the basic location of the permanent magnet, was prepared. It should be noted that all parts of the burner are considered non-magnetic and differ only in electrical conductivity. For the actual simulation, the geometry of the cathode electrode and its relative position to the volume of the magnet is important.

**Figure 6:**
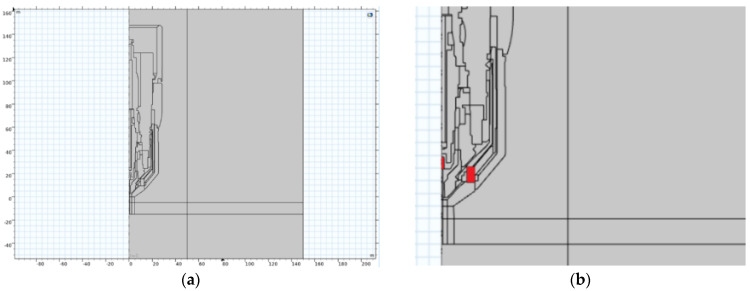
(**a**) Global axial section of a 3D model of a burner with a magnet; (**b**) detail of the burner nozzle mouth, showing the positions of the cathode and magnet (highlighted in red).

**Figure 7:**
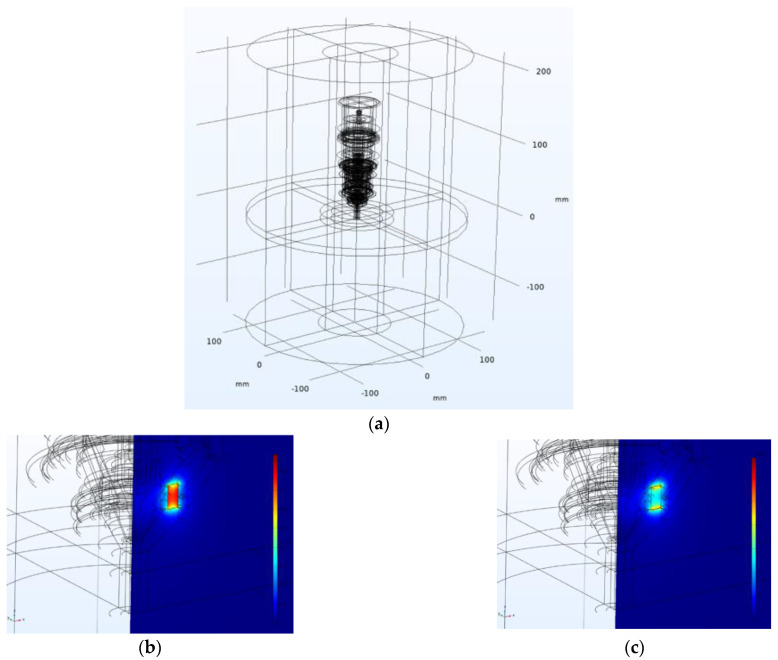
(**a**) Wireframe view of the entire 3D model; (**b**) magnetic induction *B* (T), scale 0–0.9 T; (**c**) magnetic field intensity *H* (A/m), scale 0–700 kA/m.

In this 3D simulation, the body of the arc must be replaced by a beam of electrons emitted from the cathode at a certain speed given the energy in eV and the total amount of electrons. The design of the model is shown in Figure 6a,b, with the results being obtained by calculating the distribution of the magnetic field as given in Figure 7a–c.

The motion of charged particles is solved as an interaction of the electromagnetic field which, in this case, is the interaction of a charged particle with a certain speed and the magnetic field of a permanent magnet.

**Figure 8:**
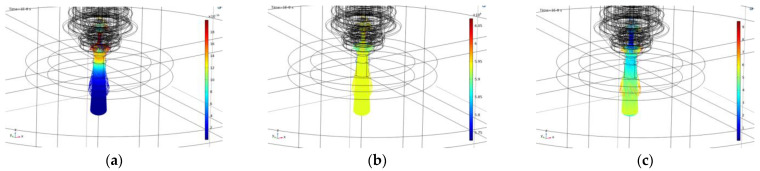
(**a**) Display of force acting on electrons *F_sum_* (N): scale 0–20 × 10^−15^ N; (**b**) particle velocity *v* (m/s): scale 0–70 × 10^5^ (m/s); (**c**) position–particle displacement *q* (mm): scale 0–10 (mm).

This 3D model, respecting the full geometry of the burner, served as a basic training model for determining the trajectories of the particles, thus determining the replacement volume of the arc hull. By tracing the electrons through the magnetic field, the results of the distribution of the electron beam were obtained as given in Figure 8a–c. Figure 8 illustrates the key physical quantities relevant to assessing the deflection of charged arc particles. Figure 8a depicts the force distribution exerted on the arc particle throughout its path in the magnetic field, measured in Newtons, with the scale indicated on the graph. Figure 8b presents the velocity distribution of the particle along its trajectory within the magnetic field of the lens, measured in meters per second (m/s), showing minimal variation. Figure 8c displays the axial deflection of the particle after traversing the magnetic field, measured in millimeters (mm), with a scale ranging from 0 to 10 mm for the given calculation scenario.

For a given configuration of the position of the cathode and magnet (height position) and the initial energy of the electrons, the electron beam is first compressed in the lens (magnet) and then significantly expanded. In principle, this is an issue of electron optics. At this moment, it is important that different positions and configurations of the permanent magnet can be used to achieve different focusing or defocusing of the electron beam. The substitute analytical calculation is quite complicated and imprecise for real geometries. Therefore, typical configurations of the electron beam, electrodes, and permanent magnet were solved in the next part of the work, where the main simulations are presented.

### 3.2. Main Simulation-Reduced 3D Burner Model for Different Geometric and Energy Variations

The basic results of the simulations corresponding to the selected geometric configurations and mutual positions of the cathode and the permanent magnet are presented, as well as the variants of the pole attachments for the given magnet and the version of the cut plate application. All simulations were performed for two-electron energy states, namely 200 eV and 500 eV.

**Figure 9:**
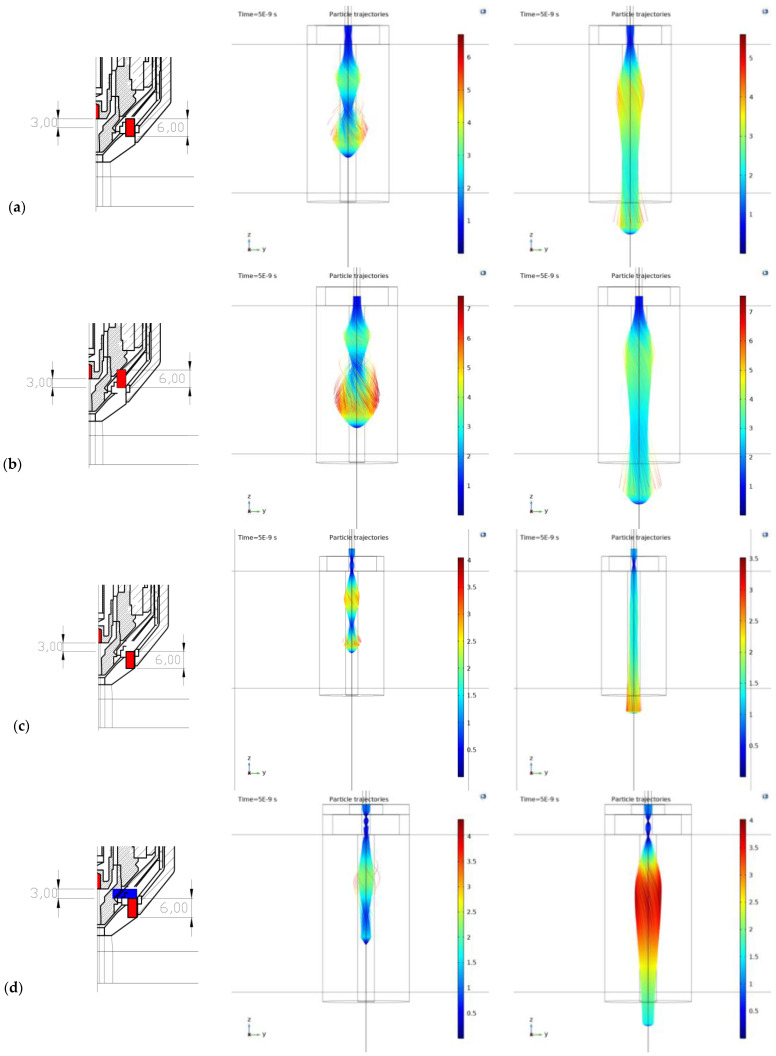

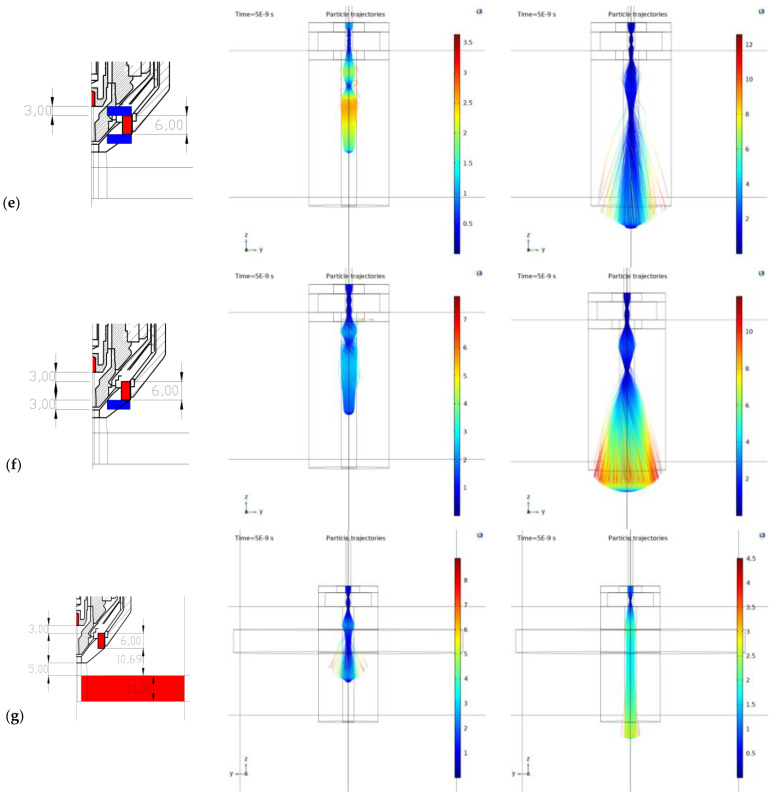

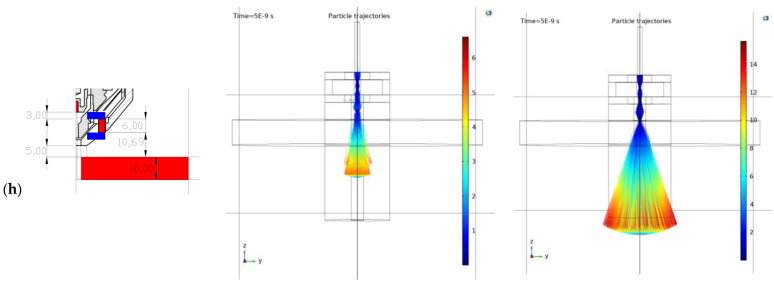
Plasma distribution (simulated particle trajectories) for configuration types: (**a**) Version 1; (**b**) version 2; (**c**) version 3; (**d**) version 4; (**e**) version 5; (**f**) version 6; (**g**) version 7; (**h**) version 8.

The key result is included in Figure 9, which contains the distribution of electron trajectories and represents the significant result of the electron beam focusing. The overview includes the following configurations:

- Version 1—the magnet itself, the electrode together with the top of the magnet—Figure 9a;
- Version 2—the magnet itself, the electrode in the middle of the magnet +3 mm down—Figure 9b;
- Version 3—the magnet itself, the electrode having moved up +3 mm—Figure 9c;
- Version 4—pole attachment only on top—Figure 9d;
- Version 5—two pole extensions (top and bottom)—Figure 9e;
- Version 6—pole attachment only below—Figure 9f;
- Version 7—the magnet itself, with the Fe plate 1 cm away from the magnet/1 cm thick—Figure 9g;
- Version 8—top and bottom pole extensions, with the Fe plate 1 cm away from magnet/1 cm thick—Figure 9h.

In versions 1 and 2, only the position of the permanent magnet changes in the plasma torch, while in versions 3–8, both the position of the permanent magnet and the arrangement of the pole extensions are changed.

The findings can be summarized as:

The application of the permanent magnet itself with a slight extension from the cathode (cathode 3 mm above the magnet) appears to be beneficial. This positioning results in the intersection of electrons at the center of the lens, minimizing subsequent scattering behind the lens, leading to a parallel/linear beam.

Conversely, when the cathode is positioned at or under the upper surface of the magnet (versions 1 and 2), a significant beam opening occurs approximately 1–1.5 cm from the bottom plane of the magnet. Despite a slight beam contraction afterwards, the pronounced initial beam opening practically eliminates beam narrowing.

Configurations involving pole extensions connected to the permanent magnet, regardless of placement (upper part, lower part, or both applied multiple times), lead to significant focusing and narrowing of the electron beam within the inner area of the lens. However, due to the scattering magnetic field, the beam is subsequently widened again. Notably, the configuration with pole extensions applied at both the top and bottom proves to be extremely unsuitable, resulting in an excessively wide electron beam when cutting a ferromagnetic plate 1 cm away from the magnet. This mirrors common usage scenarios and is more likely to be used in the field of defocusing in classic independent plasmatrons.

The magnetic field of a permanent magnet was simulated as a static magnetic field using Maxwell’s equations, simplified for this task. Essentially, it involves generating a magnetic flux created by the volume of a ring magnet, which is magnetized along its longitudinal direction. In the calculation, the magnet is specified as a magnetic hard material with the corresponding values for remanent induction (Br) and coercive force (Hc). These values, provided by the manufacturer, vary depending on the material composition and production process. In the COMSOL model, these values were converted to relative permeability and magnetization value (M). The magnet’s entire geometry and the distribution of magnetization throughout its volume were considered in the calculation. No simplifying dipole or multipole reduction were used in this simulation.

The electric arc was simplified to an electron beam, defined by a specific accelerating voltage. The total number of electrons was set to 10,000, distributed evenly on the target of a circular cathode with the kinetic energy determined by the accelerating voltage. Space-charge effects were not considered; the electrons followed independent trajectories. Relativistic corrections were not considered. The feedback of the arc/electron beam to the magnetic field of the permanent magnet was not considered.

Electrons are emitted from the cathode target into free space in a direction perpendicular to the target, directing the beam into the magnetic field of the ring magnet, where it freely enters. The area is defined by a cylindrical volume to better define and display the nozzle area, but it does not prevent the electron beam from being deflected beyond this nozzle boundary. The existence of the electron beam is limited by the calculated lifetime of the emitted electron, which defines its “range”, as the charge is not captured.

## 4. Discussion

The simulations estimate the best shape and position of the focused electron beam for various magnetic lens positions and plasma current energies. The results are intentionally presented in the target electron distribution along the path for the given and compared parameters (Figure 9). The key result maps in this figure are always structured as follows: left image—the nozzle and magnet layout, middle image—trajectory and arc distribution for 200 eV energy, and right image—trajectory and arc distribution for 500 eV energy.

The most important findings can be summarized as:

- The results demonstrate the impact of the magnet, along with the nozzle placement and configuration, providing a clear understanding of their influence and suitability. These findings, combined with the employed method, offer insights into approximating the plasma discharge focus from a cutting torch by converting the plasma current into an electron beam. This approach is applicable to specific configurations of magnet position and torch energy and can be extended to general plasma and electron beams in various applications, such as plasma welding [39], coating [40], etc.
- The initial energy and velocity of the electrons affect the curvature of their path. Higher energy results in a more evenly focused beam. Practically, lower arc currents with higher arc voltages are recommended. Additionally, the insertion height of the cathode into the top plane of the permanent magnet significantly impacts the electron beam characteristics. These conclusions are supported by the trajectory maps for each configuration.
- The beam is most uniformly focused for variant (c), where the magnet is positioned 3 mm below the cathode with a higher accelerating voltage. The deflection at the nozzle mouth here is approximately 3 mm, resulting in a parallel linear beam. In contrast, variants (e), (f), and (h), which involve specific configurations of the pole extensions, exhibit significant defocusing of the electron beam. In variant (h), the beam is deflected up to 14 mm at the nozzle mouth, with magnetic focusing having an opposite effect, causing substantial blurring and broadening of the electron beam. Other variants without pole extensions show similar behavior, with the magnitude of the effect varying due to the displacement of the beam with the magnetic field of the magnet. The key to evaluation and the overall effect lies in adjusting the initial position of the electron beam relative to the magnetic field of the magnet.
- The final configuration of the electron beam is primarily determined by the entry and exit points of the beam within the magnet’s magnetic field. Additionally, the initial energy imparted to the electrons by the accelerating voltage plays a crucial role, with significantly influence from the specific accelerating voltage applied to the electron beam and lens setup.

The use of a permanent magnet with a slight extension from the cathode (c) and (g), where the cathode is positioned 3 mm above the magnet, is the most advantageous configuration among all the analyzed variations in relative positions. The stated cathode position of exactly 3 mm above the magnet cannot be simply generalized to all variants and configurations of plasma cutters and magnet setups. The study demonstrates the principal possibility of this focusing with a clearly visible effect and provides a procedure for achieving this effect.

This position is the result of evaluating the best solution from the given solved states and variants. Parametric optimization has not been attempted yet.

The intersection of electrons occurs at the center of the lens, resulting in minimal scattering of electrons behind the lens and producing a beam that appears to be parallel or linear. This phenomenon persists both during and after cutting. Consequently, we consider this magnet design and its relative position with the cathode to be the only suitable option for practical implementation.

Only the electron beam was considered as the main carrier of the total arc current. Diffusion, recombination, and other specific properties and processes exceed the scope of this article. A simulation fully considering the arc in the form of plasma and its properties is planned. For these purposes, it is appropriate to use the Plasma module of the COMSOL package.

For now, beam divergence has only been evaluated based on the resulting trajectories. As mentioned, this task is similar to focusing an electron beam through an electron microscope, with the difference being that we are in a region with much lower accelerating voltages and therefore lower electron energies. For this reason, the resulting focusing/biasing effect of the magnetic field is much larger and more sensitive. This article is our initial study into this issue and both the issues of deflecting the arc discharge of plasma generators and deflecting the electron beam in microscopes will be addressed in further follow-up projects.

## 5. Conclusions

This article is an initial theoretical study based on finite element simulations and includes a first comparison of a plasma arc in a magnetic field with an electron beam in a magnetic field.

The results provide the first conclusions about the likely effects of typical magnet configurations and the resulting magnetic field. In addition, this work presents a practical example of simulations of charged particle tracking by magnetic fields, which can be applied in various engineering applications [41,42,43,44,45,46,47,48,49].

The computational stability was good. It was a static magnetic 3D analysis (magnetic field excited by a permanent magnet) with subsequent tracing of charged particles in the magnetic field (time-dependent task). The materials were chosen as linear, and the mesh was sufficiently dense in the necessary areas. The convergence of the calculation was smooth for the specified settings. The calculation time was approximately 30 min on a computing station with 1 CPU (8/16 cores) and 32 GB RAM memory.

The computational convergence was good, and the calculation proceeded smoothly because the problem was well and symmetrically conditioned in all aspects. Moreover, no nonlinear materials were considered here. From the solver’s point of view, it was a stationary calculation for the magnetic field of the permanent magnet and a time-dependent development for mapping electron trajectories. The solver was set to the default parameters of the electromagnetic solver.

Physical verification of the conclusions reached by computational simulations is planned in our future research. Currently, the problem is solved on a powerful computing station using COMSOL Multiphysics, as this FEM computing package handles the interconnection of various physics very well, including mechanics, heat, electricity, magnetism, fluids, and particles tracing. From the perspective of solving large problems, scalability is less optimal. However, given the size of the problems solved, the computing time and convergence on powerful computing stations are very good. Independent solutions to similar problems using other codes, such as OpenFOAM and large IT4Innovations infrastructures, are also being considered [50,51]. Moreover, a recent paper [52] highlights a fully parallel workflow for post-processing of CFD simulations. The preparation of billion-cell meshes for interactive visualization is completed in mere minutes on the Karolina cluster at the IT4Innovations National Supercomputing Center [52].

Our future research includes experimental verification and a theoretical study to provide a different perspective on the distribution of plasma generated by plasma torches. This will be based on practical experiments with DC electric arcs for various technical configurations. Additionally, future research will focus on deeper FEM simulations in this area. Moreover, we intend to utilize the sensitivity analysis and machine learning approaches introduced by [50] to evaluate how slight variations in cathode positioning impact beam stability and performance.

## Figures and Tables

**Table 1:** Constants of the Ayrton relation for copper and carbon.

	*α* [V]	*β* [V/m] ^1^	*γ* [W]	*δ* [W/m]
Cu–air	17	2000	22	18,000
C–air	40	1200	20	10,000

^1^ The quantity *β* is identical to the intensity of the electric field in the arc and is defined as the potential gradient in the direction of the arc axis.

## Data Availability

The data presented in this study are available on request from the corresponding author due to privacy and legal reasons.
